# The insect-killing bacterium *Photorhabdus luminescens* has the lowest mutation rate among bacteria

**DOI:** 10.1007/s42995-020-00060-0

**Published:** 2020-08-31

**Authors:** Jiao Pan, Emily Williams, Way Sung, Michael Lynch, Hongan Long

**Affiliations:** 1 Institute of Evolution and Marine Biodiversity, KLMME, Ocean University of China, Qingdao 266003, China; 2 Center for Mechanisms of Evolution, The Biodesign Institute, Arizona State University, Tempe, AZ 85281, USA; 3 Department of Bioinformatics and Genomics, University of North Carolina, Charlotte, NC 28223, USA

**Keywords:** Neutral evolution, Mutation accumulation, Mutation spectrum, Drift-barrier hypothesis, Lower-limit of mutation rate

## Abstract

Mutation is a primary source of genetic variation that is used to power evolution. Many studies, however, have shown that most mutations are deleterious and, as a result, extremely low mutation rates might be beneficial for survival. Using a mutation accumulation experiment, an unbiased method for mutation study, we found an extremely low base-substitution mutation rate of 5.94 × 10^−11^ per nucleotide site per cell division (95% Poisson confidence intervals: 4.65 × 10^−11^, 7.48 × 10^−11^) and indel mutation rate of 8.25 × 10^−12^ per site per cell division (95% confidence intervals: 3.96 × 10^−12^, 1.52 × 10^−11^) in the bacterium *Photorhabdus luminescens* ATCC29999. The mutations are strongly A/T-biased with a mutation bias of 10.28 in the A/T direction. It has been hypothesized that the ability for selection to lower mutation rates is inversely proportional to the effective population size (drift-barrier hypothesis) and we found that the effective population size of this bacterium is significantly greater than most other bacteria. This finding further decreases the lower-bounds of bacterial mutation rates and provides evidence that extreme levels of replication fidelity can evolve within organisms that maintain large effective population sizes.

## Introduction

Mutations are the ultimate source for genetic variation and also contribute to diseases, cell senescence, and cancer (Alfred and Knudson 1971; Davies et al. 2002). Although mutations provide the primary source for evolutionary processes, the mostly deleterious nature of mutations requires that the ability for selection to refine the mechanisms driving replication fidelity is limited by random genetic drift (Eyre-walker and Keightley 2007; Lynch et al. 2016; Sung et al. 2012a). Until now, the drift-barrier hypothesis provided the only universal explanation for mutation rate determination (Sung et al. 2016, 2012a, b).

Mutation accumulation (MA) technique combined with deep whole-genome sequencing is one of the most accurate and unbiased methods for estimating the rate and spectrum of spontaneous mutations (Halligan and Keightley 2009; Lynch et al. 2008). In a typical MA experiment involving bacteria, numerous inbred or clonal lines are cultured on rich growth media and single-colonies are transferred repeatedly. This bottlenecking process reduces the effective population size (*N*_e_, the population genetic parameter reflecting the size of an ideal population that meets all Hardy–Weinberg assumptions) and lessens the efficiency of selection, allowing all but the most deleterious mutations to drift to fixation (Bateman 1959; Mukai 1964). Using MA experiments, previous researchers have found that extremely low mutation rates exist both in unicellular eukaryotes (*Tetrahymena thermophila*—7.61 × 10^−12^ per nucleotide site per cell division (Long et al. 2016)) and bacteria (*Pseudomonas aeruginosa*—7.92 × 10^−11^ per nucleotide per generation (Dettman et al. 2016)), and both have extremely large *N*_e_ (Lynch et al. 2016).

*Photorhabdus luminescens* (Gammaproteobacteria, Enterobacteriaceae) is a facultative symbiont of nematodes that can infect insects (Boemare et al. 1993). It has a complex life-cycle involving stages of free-living, symbiosis and parasitism. *Photorhabdus luminescens* lives within the intestines of nematodes acting as a symbiont that provides luminescence and protection from pathogens (Derzelle et al. 2002; Richardson et al. 1988). It also assists the nematode in parasitizing insect larvae by releasing toxins into the blood of the insect after invasion by the nematode (Bowen et al. 1998; Daborn et al. 2001).

The genome assembly and annotation of *P. luminescens* has been published (Duchaud et al. 2003). Most previous studies have focused on mechanisms of toxicity and its use for pest control (Chalabaev et al. 2007; Derzelle et al. 2002, 2004). Other studies have explored its luminescence mechanism (Gregor et al. 2018; Winson et al. 1998). While not a human pathogen, *P. luminescens* glows at high density within human wounds, and the name “Angel’s glow” was given to the glowing of wounds of soldiers during the American Civil War (Mulley et al. 2015).

The unique biological properties and lifestyle of *P. luminescens* (symbiosis, parasitism, luminescence) raise interesting questions regarding how this organism mutates and evolves. Here, we report the findings from a 16-month *P. luminescens* mutation accumulation (MA) experiment. We estimated the genomic mutation rate and spectrum of *P. luminescens* ATCC29999 and found it has the lowest mutation rate among all bacteria studied to date.

## Results

A total of 73 *P. luminescens* MA lines were transferred every other day over the course of 16-months (232 single-cell bottlenecks over 464 days), which accounted for ~ 4517 cell divisions for each MA line. After sequencing and preliminary analysis, we removed 21 lines that were contaminants, genome library construction failures or with low sequencing coverage (minimum coverage requirement of 20 ×). The median depth of coverage for the remaining 52 lines was about 163 ×, and > 99% of the genomic sites were covered with reads in all sequenced lines (Supplementary File S1).

### Mutation rate

Using a genome that we assembled de novo, we applied established methods of mutation analysis on 52 MA lines of *P. luminescens* ATCC29999 (Lynch et al. 2008) and identified 72 base-pair substitution mutations, yielding a base-substitution mutation rate of 5.94 × 10^−11^ per nucleotide site per cell division (95% Poisson confidence intervals = 4.65 × 10^−11^, 7.48 × 10^−11^), which is the lowest bacterial mutation rate to date (Table 1, Supplementary Files S1–S3). To maintain gene function, purifying selection operates to remove mutations that arise in coding regions. In order to ensure that selection was limited in our MA experiment, we examined the distribution of mutations within non-coding and coding regions. We found that the mutation rate of *P. luminescens* ATCC29999 in non-coding regions (5.87 × 10^−11^ per nucleotide site per cell division; 95% confidence intervals: 4.49 × 10^−11^, 7.55 × 10^−11^) and coding regions (6.24 × 10^−11^; 95% confidence intervals: 3.12 × 10^−11^, 1.12 × 10^−10^) are not significantly different (Fisher’s exact test, *P* > 0.05), indicating that selection did not play a large role in this experiment.

In order to test that the extremely low mutation rate is not an artifact of the mutation analysis based on GATK, we also applied the consensus approach, which has a low false-negative rate (Farlow et al. 2015; Keightley et al. 2015). The number of mutations detected by the GATK method is about 93% of that of the consensus method. The base-pair substitution mutation rate from the consensus method is 6.34 × 10^−11^ per nucleotide site per cell division vs. 5.94 × 10^−11^ (95% confidence interval: 4.65 × 10^−11^, 7.48 × 10^−11^) using the GATK method. Thus, the extremely low mutation rate of *P. luminescens* does not result from false negatives. The false positive rate of the mutation analysis, also used in previous studies, was < 1% with either Sanger sequencing or Miseq re-sequencing of mutation calls. Consequently we did not implement this step for the bacterial MA in the present study (Farlow et al. 2015; Long et al. 2015; Nguyen et al. 2020).

There were in total four insertion and six deletion mutations across all lines, yielding an indel mutation rate of 8.25 × 10^−12^ per site per cell division (13.89% of the base-pair substitution rate; 95% confidence intervals: 3.96 × 10^−12^, 1.52 × 10^−11^), the lowest indel mutation rate in all studied bacteria to date. This finding is consistent with a ~ 10-fold reduction between base-substitution mutation rates and indel mutation rates in bacteria (Sung et al. 2016).

### Mutation spectrum

**Fig. 1:**
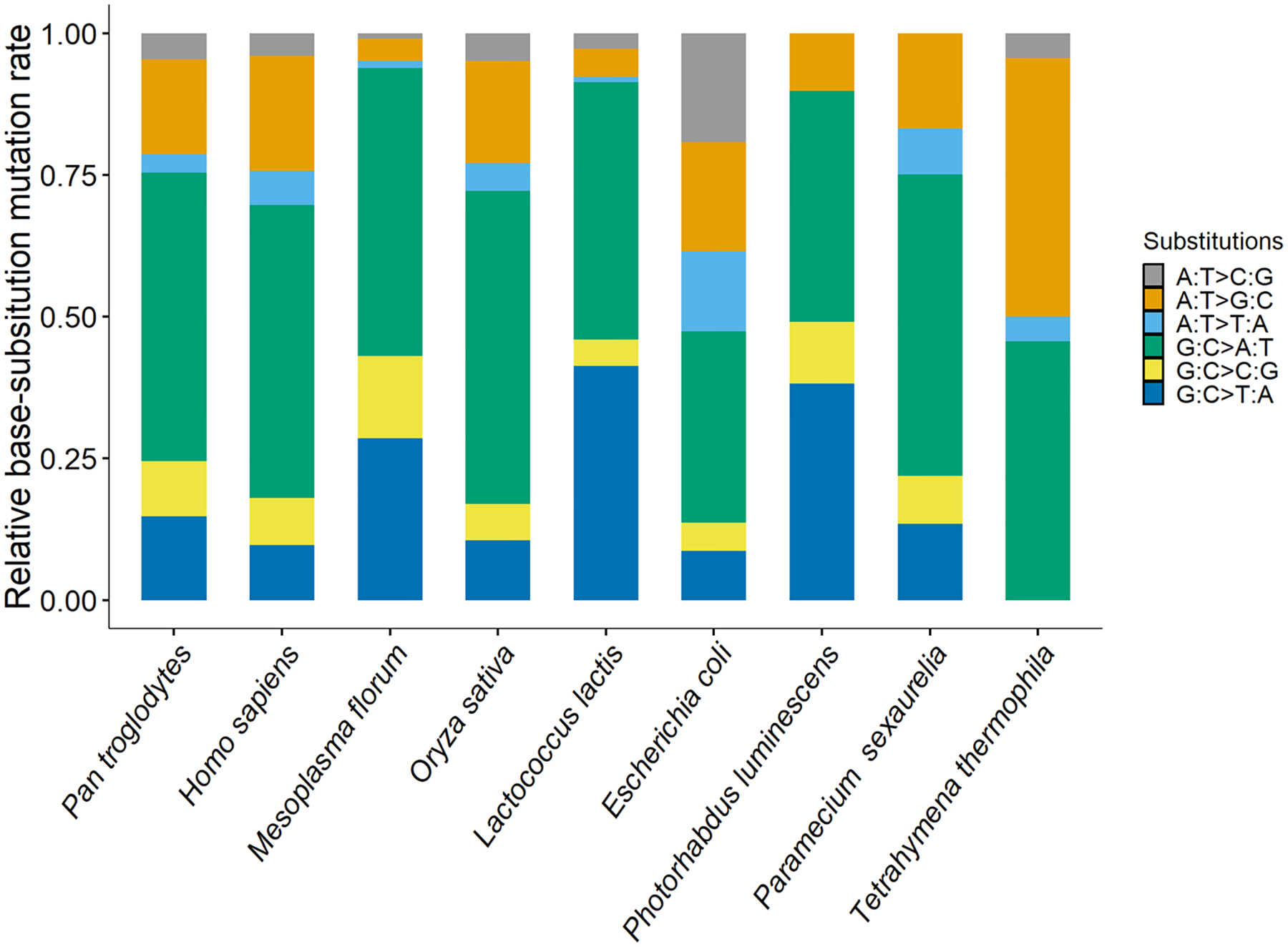
The relative base substitution mutation rates of different organisms, which cover almost the full range of mutation rates of repair-functional organisms (Long et al. 2016, 2018a; Lynch et al. 2016). From left to right, the mutation rates are ordered from high to low and belong to *Pan troglodytes verus*, *Homo sapiens* CEU, YRI, Iceland, *Mesoplasma florum* L1, *Oryza sativa*, *Lactococcus lactis* DSMZ20481, *Escherichia coli* K-12 MG1655, *Photorhabdus luminescens* ATCC29999, *Paramecium sexaurelia* and *Tetrahymena thermophila*, respectively

**Fig. 2:**
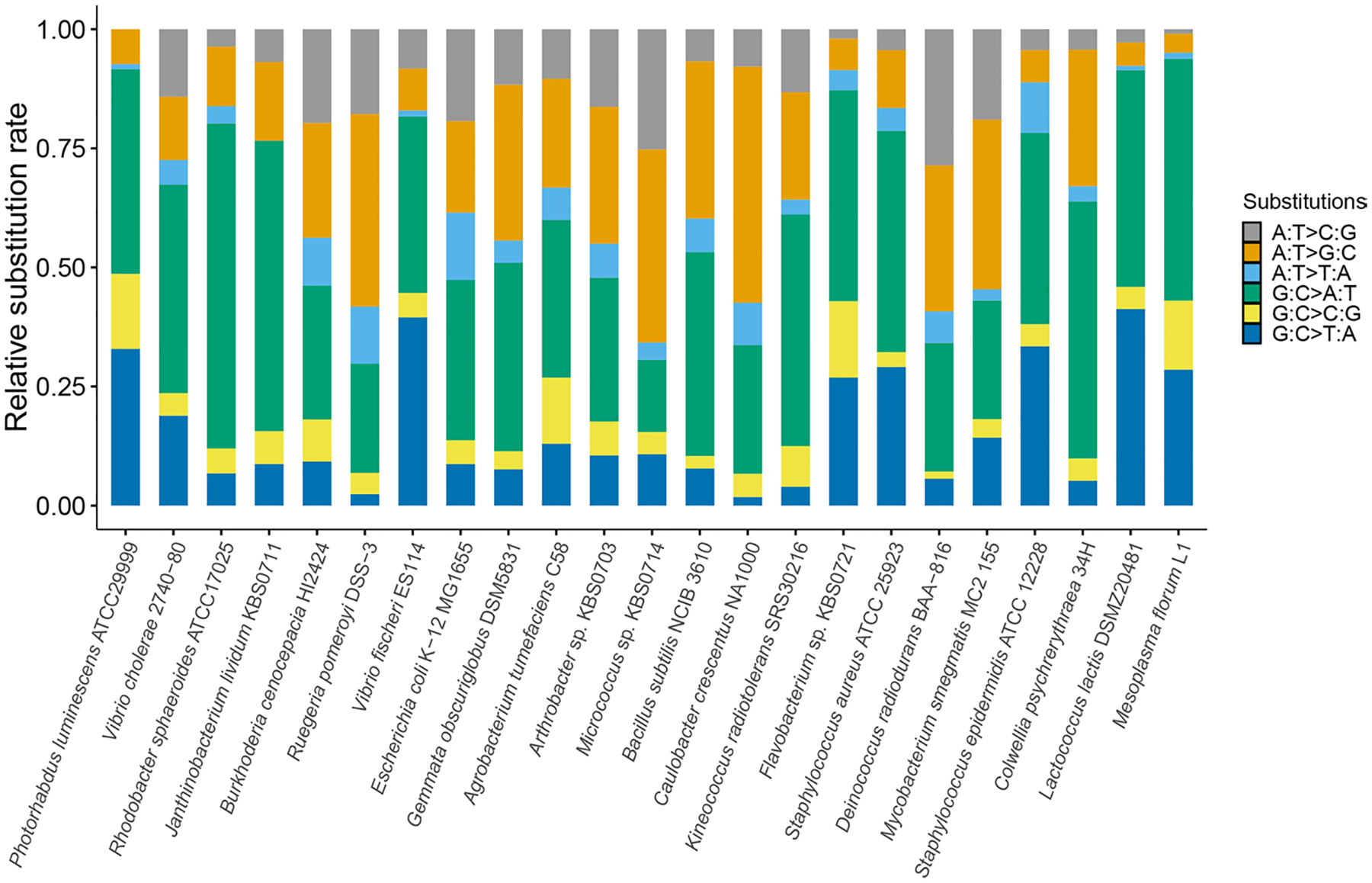
The relative base substitution mutation rates of *P. luminescens* and other bacteria (Long et al. 2018a). From left to right, the mutation rates are ordered from low to high

A/T→G/C and G/C→A/T mutation rates were 1.00 × 10^−11^ (0.40–2.07 × 10^−11^) and 1.03 × 10^−10^ (0.77–1.35 × 10^−10^) respectively, yielding a mutation bias to A/T of 10.28, one of the highest in bacteria (Tables 1, 2; Figs. 1, 2). This strong A/T bias is driven by the dominating G:C→A:T transition rate 5.84 × 10^−11^ and G:C→T:A transversion rate 4.47 × 10^−11^, in contrast to the mutation rates in the G/C direction: A:T→G:C transition rate 1.00 × 10^−11^ and zero A:T→C:G transversions.

We also estimated the expected equilibrium A + T content of the genome to be 91.14%, the highest in bacteria (Long et al. 2018c), while the A/T content of the reference genome is 57.59%. Hypotheses regarding the evolution of genome-nucleotide composition suggest that lower than expected genomic A/T content from mutation alone could be caused by natural selection and gene conversion against mutations in the A/T direction (Long et al. 2018c). Due to the extremely low mutation rate, only three types of transversions were observed (Table 2): G:C→T:A (23), G:C→C:G (11), and A:T→T:A (1). The transition to transversion ratio (ts/tv) of *P. luminescens* is 1.06, similar to that of many previously-reported wild-type bacteria.

### The effective population size

To estimate the effective population size of *P. luminescens*, we aligned the genome sequences of 12 *P. luminescens* strains available in NCBI, including the draft genome of *P. luminescens* ATCC29999 assembled here (see “Materials and methods”). We found that the nucleotide diversity at four-fold degenerate sites (*θ*; four-fold degenerate sites: 280,141, SNPs: 49,343) was 8.31 × 10^−2^. Using mutation rate estimates from this study, we calculated the effective population size (*N*_e_) of *P. luminescens* to be approximately 7.00 × 10^8^, one of the largest reported among all bacteria (Lynch et al. 2016) (Table 1).

## Discussion

Using MA experiments combined with deep whole-genome sequencing, we calculated the mutation rate of *Photorhabdus luminescens* ATCC29999, which is 5.94 × 10^−11^ per site per cell division. This is the lowest known measurement of mutation rates in bacteria. According to the drift-barrier hypothesis (Sung et al. 2012a), the ability for selection to refine the genetic mechanisms responsible for DNA fidelity is positively correlated with effective population size. Thus, organisms with large effective population sizes have extremely efficient DNA repair and synthesis, and consequently extremely low mutation rates. *Photorhabdus luminescens* has the largest estimate of *N*_e_ seen in bacteria and this may arise from high genome plasticity and widespread horizontal gene transfer observed in this bacterium (Gaudriault et al. 2008; Tounsi et al. 2006). It is noteworthy that estimates of effective population size are very dependent on diversity estimates from sequenced strains, and including additional representative strains of *P. luminescens* when estimating nucleotide diversity may improve estimates of *N*_e_. Nevertheless, our findings are consistent with the theoretical framework established by the drift-barrier hypothesis regarding the evolution of mutation rate across the tree of life.

*Photorhabdus luminescens* mutations are more strongly A/T-biased than 87% of studied bacteria (Supplementary Table S1 in Long et al. (2018c); Table 1). This A/T bias is caused by the extremely low mutation rate in the G/C direction, especially 0 A:T→C:G transversion rate (Table 2). Besides regular oxidative repair systems such as *mutT*/*mutH* homologs (see the genome annotation in Supplementary File S4), *P. luminescens* can also express luciferase, which is an oxygen scavenger and thus reduces free 8-oxo-guanines in the cytoplasm (Ffrench-Constant et al. 2003). Unlike 8-oxo-guanines in the DNA strands, free 8-oxo-guanines in the nucleotide pool of the cytoplasm could be incorporated into newly synthesized DNA strand and pair with adenines during DNA replication. In the next replication round, after the incorporated 8-oxo-guanine is repaired to guanines and becomes a template, the original A:T pair becomes a C:G pair at the site, i.e., A:T→C:G transversion (Lynch 2007). We thus speculate that luciferase could be an important factor in decreasing the A:T→C:G transversion rate. If this is the case, other bacteria with luciferase should also show low A:T→C:G transversion rates. We thus blasted the NCBI gene database using the *P. luminescens* luciferase sequence as a query and also required the hit species to have resolved genomic mutation spectra. We found one bacterium, i.e., *Vibrio cholerae* (luciferase blast results: 80% amino acid sequence identity with 95% coverage; BPS rate: 1.07 × 10^−10^), which does indeed have a low A:T→C:G transversion rate of 0.33 × 10^−10^ (re-analyzed from Dillon et al. (2017) and Sung et al. (2016); Table 1). Despite this, mutation data of other luminescent species are needed in order to demonstrate a definitive association between luciferase function and low A:T→C:G transversion rates in bacteria, since many pathways could be involved in decreasing this type of mutations and the relative potency between these pathways and luciferase remains to be tested.

With limited data points in a subset of model microbes, Drake (1991) proposed a constant 0.0033 mutation per genome per generation for DNA-based microbes. However, recent MA studies have revealed that bacteria exhibit a large variation in mutation rates with significantly higher and lower mutation rates per genome per generation than previously expected (Sung et al. 2012a) (Table 1). The present MA experiment on *P. luminescens* reveals a new lower limit for bacterial mutation rate suggesting that DNA fidelity can reach extreme refinement in prokaryotes. It is unlikely that *P. luminescens* has achieved the lowest limit attainable for bacteria, and it remains to be seen how low mutation rates can go (Sniegowski and Raynes 2013). Mutation rates from MA using more than one strain per species would be more representative since rates are known to vary within a species (Hamilton et al. 2017; Morgan et al. 2014). Unfortunately, we were only able to include one strain of *P. luminescens* due to the limitation of research resources and the large number of species that needed to be investigated in this project. The findings of the present study should also inspire investigations of the highest mutation rate limit. Previous studies show that if there is one mutation in one cell division in asexual populations, then mutational meltdown would occur and kill the organism (Eigen 1971; Lynch et al. 1993). Most DNA mismatch repair-deficient organisms have ~ 0.1 to 0.3 mutation per cell division per genome (Long et al. 2018b), which leaves plenty of scope for exploring high mutation rates and the mutagenesis mechanisms involved. These studies would help to understand the long-term evolution of mutation rates, factors affecting genomic stability, and mutagenesis patterns common in natural organisms and diseases.

## Materials and methods

### Cell lines and transfers

In total, 73 MA lines from a single progenitor cell of *Photorhabdus luminescens* ATCC29999 (obtained from ATCC) were initiated and passed through single-cell bottlenecks every other day on nutrient agar plates at 30 °C. Every month, single colonies from 10 randomly selected MA lines were cut from the nutrient agar plates and serially diluted to count colony forming units (CFU). The mean number of cell divisions that took place to form a colony from a single cell was estimated by log_2_(CFU). The total number of cell divisions of each MA line is the product of the mean (19.5) of all cell division estimates and the total number of transfers for each line. On average, each MA line experienced 232 transfers. The total time taken to complete the process of transferring each MA line was 464 days. The total number of cell divisions per MA line was 4517. We removed one MA line (photo41) due to cross contamination with photo42 and photo47, which was detected after genome sequencing. One line (photo49) only having part of the genome sequenced was also excluded. Other lines were removed from the final mutation analysis because of genome library construction failure or low coverage (< 20 ×). Eventually, 52 cell lines were used in the final mutation analysis.

### DNA extraction, library construction, and genome sequencing

We extracted DNA from the final MA lines using the Wizard^®^ Genomic DNA Purification Kit (Promega, USA) and constructed libraries using the Nextera^®^ DNA Library Preparation Kit (Illumina, USA). DNA libraries with insert size ~ 300 bp were sequenced by an Illumina HiSeq2500 sequencer at Hubbard Center for Genome Studies, University of New Hampshire, with 2 × 150 bp rapid run.

### de novo assembly and annotation of *P. luminescens* ATCC29999 genome

Initially, we used the reference genome of *Photorhabdus luminescens* ATCC29999 (GenBank accession number: GCA_900102985.1; with the minimum size of 1000 bp) for preliminary mutation analysis and obtained a base-pair substitution rate of 4.39 × 10^−11^ per nucleotide site per cell division. This is comparable to the mutation analysis using the de novo assembled genome (see below). However, we found the breadth of coverage of the MA lines was lower than 70%, i.e., proportion of the genome covered by reads. This was possibly caused by over-filtering when the reference genome was assembled, indicated by the low number of scaffolds in the assembly. We thus used Unicycler -v0.4.8 (Wick et al. 2017) to assemble a new draft genome of *P. luminescens* ATCC29999 to analyse the mutations using the paired-end reads of one MA line (photo7), which did not have any mutations after mapping to the above NCBI reference genome. It is noteworthy that NCBI does not allow contigs in assemblies shorter than 200 bp to be uploaded whereas we needed to retain these in order to detect all mutations, so we could not upload this assembly to NCBI. We therefore present the assembly and annotations in the supplementary files. The draft genome (Supplementary File S2) has 280 scaffolds with a total size of 5,171,217 bp, GC content: 42.39%, N50: 92,679 bp and no gaps. Of the 280 scaffolds, 42 are shorter than 200 bp. The total length of these short contigs is about 0.12% of the genome size, mean sequencing depth of coverage 151, and 89% of them are covered with reads. Extremely short contigs might cause false negatives, due to mapping failure of reads. A comparison of the mutation rates of the two genomes showed that these extremely short contigs did not bias the mutation rate. For annotation, we used RAST (https://rast.theseed.org) (Aziz et al. 2008). The annotation file (Supplementary File S4) has 5,240 predicted protein-coding genes, with the average length of 854 bp. We used BUSCO-3.1.0 (Seppey et al. 2019) to assess the draft genome using the database of bacteria_odb9 and found that the coverage rate of complete universal single-copy orthologs in the genome was 99.3%, indicating high completeness of the assembly. All analyses reported in this study were based on the draft genome we assembled for *P. luminescens* ATCC29999.

### Mutation analyses

We first used Trimmomatic 0.32 (Bolger et al. 2014) to trim the library adapters for the raw reads and BWA mem -ver. 0.7.12 (Li and Durbin 2009) to map the reads to the draft genome of *P. luminescens* ATCC29999. We used samtools-1.3.1 (Li et al. 2009) to transform sam files to the bam format. Duplicate reads were removed using picard-tools-2.5.0, and SNP/indel variants were called using HaplotypeCaller in GATK-4.0 using standard hard filters across all 52 MA lines (McKenna et al. 2010; Van der Auwera et al. 2013). We also visually verified every single mutation using IGV-2.4.10 (Robinson et al. 2011).

The mutation rate was calculated using the following formula:

$$\mu =\frac{M}{\sum_{1}^{n}N\times T},$$

where *μ* is the mutation rate, *M* is the total number of mutations in all MA lines, *n* is the total number of lines, *N* is the analyzed sites in one line, and *T* is the number of cell divisions that took place during the mutation accumulation experiments in a single line. The 95% Poisson confidence intervals of the mutation rate were calculated by the cumulative Poisson probability approximated by the binomial distribution. We also used the consensus approach to perform mutation analyses to test our false negative rate (Sung et al. 2012a).

We calculated the mutation bias in the A/T direction *m* by *m* = *μ*_G:C→A:T+G:C→T:A_/*μ*_A:T→G:C+A:T→C:G_, and the transition to transversion ratios (ts/tv) of *P. luminescens* with the following formula:

$$\frac{\text{ts}}{\text{tv}}=\frac{\sum_{1}^{n}\text{transitions}}{\sum_{1}^{n}\text{transversions}}.$$

We calculated the expected equilibrium A+T content of the genome *p* using the following formula:

$$p=\frac{v}{u+v},$$

where *u* is the mutation rate in the G/C direction, i.e., A/T→G/C, and *v* is the G/C→A/T mutation rate (Lynch 2007).

### Effective population size

The genome of *P. luminescens* ATCC29999 assembled in this study and those of another 12 *P. luminescens* strains with > 95% sequence identity in the 16S rRNA gene sequence were used to calculate the nucleotide diversity at four-fold degenerate sites (Table 3; *P. luminescens* DSM 3368 was not used as it was actually a synonym of ATCC29999, as shown in the ATCC website). These 13 genomes were aligned using Mugsy (Angiuoli and Salzberg 2010) and the average pairwise genetic distance at four-fold degenerate sites (*θ*) was calculated using:

$$\theta =\frac{n}{n-1}\times \left(1-\sum p^{2}\right),$$

where *p* is the average allele frequency of nucleotides per four-fold degenerate site between DNA sequences in all possible pairs of all strains, *n* is the number of strains, *n*/(*n* − 1) is the calibration factor. The effective population size (*N*_e_) of *P. luminescens* was then calculated using the following formula:

$$N_{\text{e}}=\frac{\theta }{2\mu }.$$

### Data availability and statistics

Illumina reads of all MA lines in the final analysis were deposited in NCBI SRA (BioProject Number: PRJNA376572). All statistical tests and plotting were performed in R-3.5.1 (R Development Core Team 2012). All genome sequence analyses were done using the IEMB-1 computation cluster of Institute of Evolution and Marine Biodiversity, Ocean University of China.

## Supplementary Material

S1

S2

S3

S4

## Figures and Tables

**Table 1:** Genome size, base substitution rate (*μ*), mutation bias in the A/T direction (*m*, *m* = *μ*_G:C→A:T+G:C→T:A_/*μ*_A:T→G:C+A:T→C:G_) and the effective population size (*N*_e_) of different species of bacteria

Species	Genome size (Mb)	*μ*	*μ*_A:T > C:G_	*m*	*N*_e_
*Agrobacterium tumefaciens* C58	5.67	2.92	0.60	1.38	3.42
*Bacillus subtilis* NCBI 3610	4.29	3.28	0.44	1.27	0.61
*Burkholderia cenocepacia* HI2424	7.70	1.33	0.49	0.85	2.47
*Escherichia coli* K-12 MG1655	4.64	2.23	0.86	1.10	1.80
*Photorhabdus luminescens* ATCC29999	5.17	0.59	0.00	10.28	7.00
*Pseudomonas aeruginosa* PA14	6.58	0.79	0.21	1.52	2.10
*Pseudomonas fluorescens* SBW25	6.72	0.93	0.29	1.17	-
*Staphylococcus aureus* ATCC25923	2.81	4.38	0.48	4.57	-
*Vibrio cholerae* 2740–80	3.95	1.07	0.33	2.30	4.78

Except for *P. luminescens* ATCC29999, data are from Dettman et al. (2016), Long et al. (2018c), and Lynch et al. (2016). *μ* and *μ*_A:T > C:G_ are in units of × 10^−10^ per site per cell division, *N*_e_ is in 10^8^

**Table 2:** Mutation spectrum of *P. luminescens* ATCC29999

Substitutions	Count	*μ*	CI
Transitions			
G:C→A:T	30	5.84	3.937, 8.330
A:T→G:C	7	1.00	0.403, 2.065
Transversions			
A:T→T:A	1	0.14	0.004, 0.798
G:C→T:A	23	4.47	2.836, 6.712
A:T→C:G	0	0	0.000, 0.528
G:C→C:G	11	2.14	1.068, 3.828
Insertions	4	0.33	0.090, 0.845
Deletions	6	0.49	0.182, 1.077

Mutation rates (*μ*) are in units of × 10^−11^ per site per cell division CI 95% Poisson confidence intervals

**Table 3:** Names and genome accession numbers of different strains of *Photorhabdus luminescens* and 16S rRNA gene sequence identity with ATCC29999

Strain name	GenBank assembly accession	Identity (%)
HIM3	GCA_002204205.1	99.61
MEX47–22	GCA_004348775.1	99.53
Caborca	GCA_006239335.1	95.89
NBAII H75HRPL105	GCA_000826725.2	95.58
H1	GCA_002968995.1	95.58
LN2	GCA_000767775.1	95.50
H5	GCA_002969055.1	95.50
BA1	GCA_000612035.1	95.50
NBAII HiPL101	GCA_000798635.2	95.43
NBAII Hb105	GCA_000931955.2	95.43
H4	GCA_002969005.1	95.43
H3	GCA_002968975.1	95.43
